# Comparison of conventional and unconventional obesity indices associated with new-onset hypertension in different sex and age populations

**DOI:** 10.1038/s41598-023-34969-0

**Published:** 2023-05-13

**Authors:** Xueyao Zhang, Guangxiao Li, Chuning Shi, Yichen Tian, Linlin Zhang, Hongyu Zhang, Yingxian Sun

**Affiliations:** 1 Department of Cardiology, First Hospital of China Medical University, 155 Nanjing North Street, Heping District, Shenyang, 110001 China; 2 Department of Medical Record Management, First Hospital of China Medical University, Shenyang, China

**Keywords:** Cardiology, Endocrinology, Health care, Medical research, Risk factors

## Abstract

We aimed to compare the relationship between hypertension and obesity-related anthropometric indices (waist circumference [WC], waist-height ratio, waist-hip ratio [WHR], and body mass index; unconventional: new body shape index [ABSI] and body roundness index [BRI]) to identify best predictors of new-onset hypertension. The study included 4123 adult participants (2377 women). Hazard ratios (HRs) and 95% confidence intervals (CIs) were determined using a Cox regression model to estimate the risk of new-onset hypertension with respect to each obesity index. In addition, we assessed the predictive value of each obesity index for new-onset hypertension using area under the receiver operating characteristic curve (AUC) after adjusting for common risk factors. During the median follow-up of 2.59 years, 818 (19.8%) new hypertension cases were diagnosed. The non-traditional obesity indices BRI and ABSI had predictive value for new-onset hypertension; however, they were not better than the traditional indexes. WHR was the best predictor of new-onset hypertension in women aged ≤ 60 and > 60 years, with HRs of 2.38 and 2.51 and AUCs of 0.793 and 0.716. However, WHR (HR 2.28, AUC = 0.759) and WC (HR 3.24, AUC = 0.788) were the best indexes for predicting new-onset hypertension in men aged ≤ 60 and > 60 years, respectively.

## Introduction

Obesity is a major global public health issue that increases the risk of type 2 diabetes, fatty liver disease, hypertension, cardiovascular disease, and multiple cancers^1^. Alongside urbanization, changes in food supply and eating habits, and a reduction in physical activity, the obesity rate more than tripled in low socio-economic populations such as the Middle East, Oceania, and China^2^. Currently, more than one-third of the global population is overweight or obese. At least 60% of obese patients live in developing countries, and the prevalence of hypertension and obesity-related cardiac metabolic disorders is rapidly increasing^3^. To date, there has been insufficient focus on the impact of obesity with hypertension incidence in rural China.

Hypertension is a known variable risk factor for cardiovascular disease, with obesity an independent risk factor for hypertension^4^. In addition, people with obesity are 3.5 times more likely to suffer from hypertension, and 60% of hypertension cases are attributed to an increase in fat storage. Previous data showed the prevalence of hypertension in obese individuals with a body mass index (BMI) > 30 kg/m^2^ was 42.5%, significantly higher than 15.3% in non-obese individuals^5^. The conventional anthropometric indicators that define obesity include BMI, waist circumference (WC)^6^, waist-height ratio (WHtR), and waist-hip ratio (WHR)^7^. Unconventional new indicators proposed in recent years include a body shape index (ABSI)^8^, and body roundness index (BRI)^9^.

Epidemiological studies evaluated the relationship between conventional anthropometric obesity indices and blood pressure (BP). However, the results are inconsistent and the optimal index unconclusive. Zhang et al. of a recent cross-sectional study concluded that WC and BMI were related to hypertension, and an additive interaction between WC and BMI existed^10^. A prospective cohort study returned comparable findings^11^. However, some authors question the predictive value of BMI. It cannot distinguish between fat and muscle mass nor reflect the individual fat distribution^12^. Some studies suggested WC, WHtR, and WHR were alternative indicators of obesity. However, other studies did not concur with WHR as a predictor of hypertension^13^. In terms of unconventional obesity indicators^14^, BRI was found to be able to determine whether cardiovascular disease (CVD) existed, while ABSI cannot. However, the ability of BRI to recognize CVD was not better than BMI and WC. Other studies found ABSI could predict premature death by expressing the additional risk from high WC, using a simple formula^8^. At present, the predictive value of unconventional obesity-related indicators for hypertension is unelucidated.

Preliminary studies inferred that inconsistency in the research of optimal conventional obesity indicators may be related to nationality, eating habits, climate differences, and baseline BP. Moreover, the relationship between obesity and hypertension may also be affected by sex and age^15^, yet previous studies were mostly cross-sectional and not cross-grouping by age and sex. The relationship in different sex and age groups in the Chinese population between obesity indicators and the incidence of hypertension is not entirely clear, and the relationship between unconventional obesity indicators (BRI and ABSI) and hypertension has not yet been explored. Compared to in urban areas, rural areas in China have a high incidence rate of hypertension and even cardiovascular diseases. In 2018, the incidence of hypertension in urban areas was 25.7%, while in rural areas it was 29.4%^16^. Moreover, the relative incidence rate in rural areas was higher, but the control rate was lower. Previous studies had found that the incidence rate of hypertension in rural areas in Northeast China was as high as 50%, but the control rate was only 5.5%^17^. Secondly, medical care in rural areas was relatively backward^18^, and the burden of hypertension was relatively greater. Therefore, it is necessary to pay more attention to the prevention of hypertension before its onset^19^. In addition, the obesity incidence rate in China is currently increasing, and it is expected to increase to 70.5% by 2030. Although the urban–rural gap is narrowing, it is possible to achieve in the future a reversal of this trend of incidence in rural areas surpassing that in urban areas^20^. Therefore, to address this urgent issue that China needs to resolve, this study aimed to explore whether unconventional and conventional obesity indicators can predict new-onset hypertension in a prospective cohort in rural areas of Northeast China. We also aimed to compare the predictive value of various obesity-related anthropometric indices for new-onset hypertension prediction among different sex and age groups.

## Materials and methods

### Study design and data collection

The Northeast China Rural Cardiovascular Health Study (NCRCHS) was conducted from January 2012 to August 2013. The study design of this community-based prospective cohort study was previously described^21^. First, three counties (Dawa, Zhangwu, and Liaoyang) were selected from the eastern, southern, and northern regions of Liaoning Province. In the second stage, one town was randomly selected from each county (three towns total). In the third stage, 8–10 rural villages were randomly selected from each town (26 in total). Participants who were pregnant, had malignancies, or had mental disorders were excluded. In total, 11,956 permanent residents aged ≥ 35 years in each village were invited to participate. The response rate was 89.5%. Overall, 10,700 participants agreed and qualified to participate in our follow-up study, and baseline information on each participant was collected.

**Figure 1:**
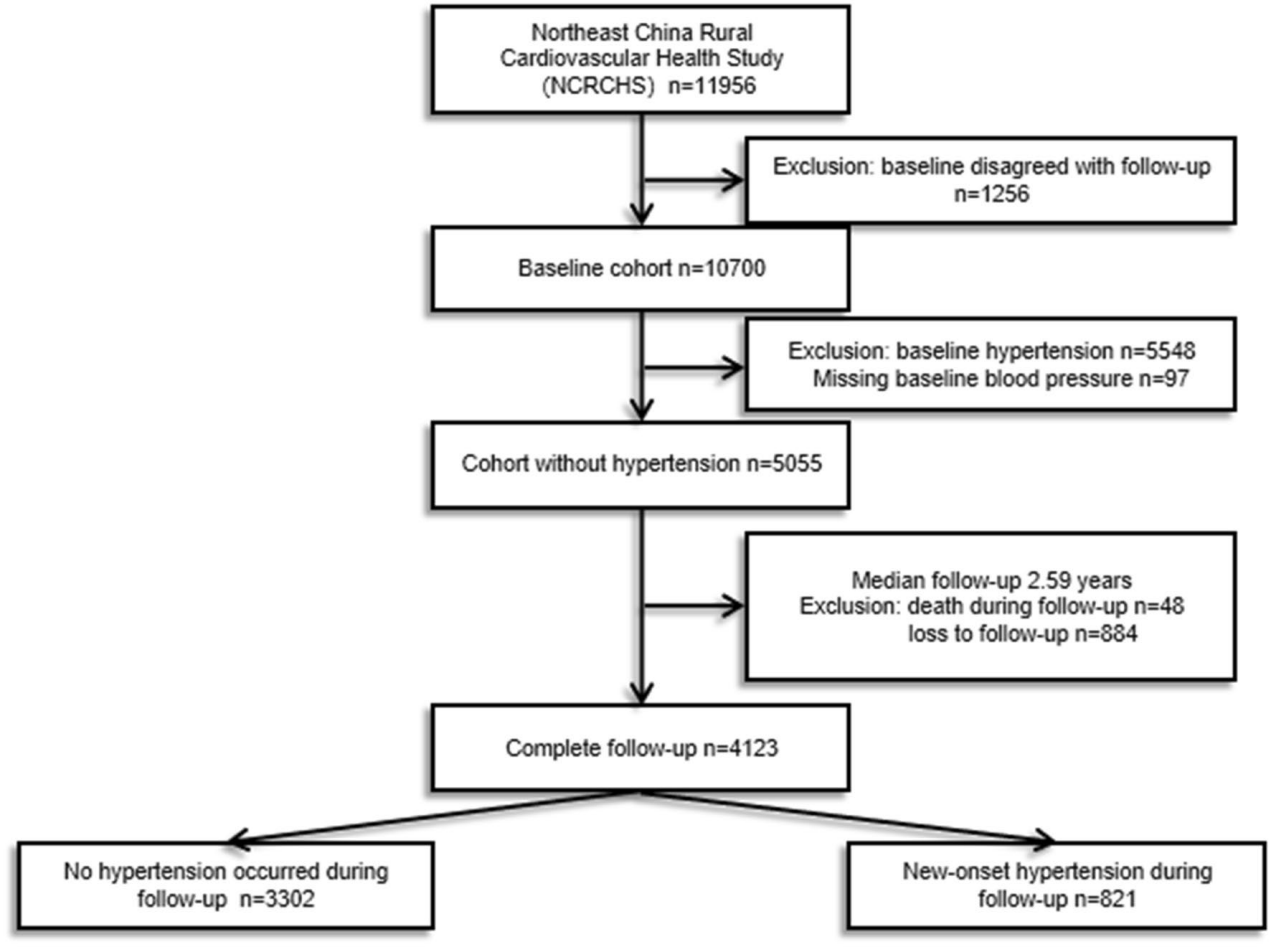
Flow chart of subject inclusion and exclusion.

In this study, trained researchers from China Medical University interviewed each participant using a standardized questionnaire, face-to-face interview, physical examination, and blood biochemical test. Clinical, demographic, and lifestyle information of all participants was collected. Exclusions included 97 people due to incomplete baseline BP data, 5548 people with baseline hypertension, 48 deaths in the median follow-up of 2.59 years, and 884 people without follow-up BP data information. Finally, we collected data from 4123 participants, including 2377 women, to assess the correlation between BMI, WC, WHR, WHtR, BRI, ABSI, and new-onset hypertension (Fig. 1).

### Exposure and outcome ascertainment

Anthropometric indicators were measured according to standard procedures. We used a standard rangefinder to measure the barefoot height of participants. The subjects wore light indoor clothes when using the electronic scale to measure weight. The WC was measured with an inelastic anthropometric belt at about belly level at the midpoint between the lowest edge of the ribs and the level of the anterior superior iliac crest. We measured twice at the end of normal exhalation and recorded the average value. The hip circumference was measured at the maximum protrusion of the gluteal muscle. Readings were recorded to the nearest 0.1 cm and 0.1 kg. BMI was calculated by dividing an individual's weight in kilograms by the square of their height in meters. Considering the small stature of subjects in rural China, according to the criteria of the China Obesity Working Group (divided into < 18.5, 18.5–23.9, 24.0–27.9, and ≥ 28 kg/m^2^), a BMI > 28 kg/m^2^ was defined as obesity^22^. WHR was calculated by dividing WC by hip circumference, and WHtR was calculated by dividing WC by height. According to World Health Organization standards, the central obesity of men was WC ≥ 90 cm or WHtR ≥ 0.5 or WHR ≥ 0.9; that of women was WC ≥ 80 cm or WHtR ≥ 0.5, or WHR ≥ 0.85^7^. ABSI and BRI were calculated as follows^8,9^:

$${\text{ABSI }} = {\text{ WC}}\div(BMI^{2/3}\times Height^{1/2})$$

$${\text{BRI }} = {364}.{2} - {365}.{5 } \times \sqrt {1 - (WC^{2} \div (\pi \times {\text{Height}})^{2} )} .$$

Specific consulting rooms were used at the recruitment and follow-up sites. During BP measurement, researchers from the project team used HEM-907, a standard electronic blood pressure monitor (Omron, Japan), to evaluate the right arm BP after participants had rested in a sitting position for 10 min. Each measurement interval was at least 30 s. The average of the three closest readings was taken as the BP value. All participants were instructed not to exercise, smoke, or drink irritant-containing beverages (e.g., tea, coffee, alcohol) before BP measurement. Participants also underwent detailed cardiovascular examinations in 2015 which included face-to-face visits and on-site blood pressure measurement review. Participants were also required to report on blood pressure measured at home or village clinics and on related diagnostic and treatment processes. The standard parameters for defining hypertension were a systolic blood pressure ≥ 140 mmHg and/or a diastolic blood pressure ≥ 90 mmHg, or the use of anti-hypertension drugs^23^.

### Covariates

Information on demographic characteristics (e.g., age, sex, marital status, education level, income level) and behavioral measures (smoking, exercise, drinking, physical activity intensity) was obtained through standardized questionnaires, and the measures were carried out in strict accordance with the national standard for basic public health services (2011). All participants were required to collect pre-elbow venous blood on an empty stomach (more than 12 h) in the morning. After it stood at room temperature for half an hour, the blood sample was centrifuged at 3000 rpm for 5 min. The supernatant was collected and stored at − 20 °C and transferred to the laboratory within 4 h for immediate testing. The Abbott Diagnostics C800i automatic analyzer (Abbott Laboratories, Abbott Park, IL, USA) was used for routine index determination. Blood lipid abnormalities were classified according to the NCEPATPIII standards and liver function cutoff values according to previous literature standards^24,25^: high low-density lipoprotein cholesterol (LDL-C) was defined as ≥ 4.14 mmol/L; low high-density lipoprotein cholesterol (HDL-C) was defined as < 1.04 mmol/L; high total cholesterol (TC) was defined as ≥ 6.22 mmol/L; and high triglyceride (TG) was defined as ≥ 2.27 mmol/L. Increased aspartate transaminase (AST) was defined as > 35 U/L; high alanine transaminase (ALT) was defined as > 40 U/L; high uric acid (UA) for males was ≥ 420 μmol/L and for females was ≥ 360 μmol/L^26^; and high white blood cell (WBC) count was defined based on the laboratory reference cutoff as greater than 10 × 10^9^.

### Statistical analysis

The Shapiro–Wilk and Kolmogorov–Smirnov tests were used to test the normality of the dataset. All continuous variables are described as mean ± standard deviation (SD) after the normality test, while categorical variables are described as percentages. The Student’s *t*-test and Chi-square test were used to estimate the differences between continuous variables and categorical variables in different groups. This study estimated the following obesity-related variables: BMI, WC, WHR, WHtR, BRI, and ABSI. Hazard ratios (HRs) and 95% confidence intervals (CIs) were used to estimate the risk of new-onset hypertension for each obesity index (all binary variables) with the help of the Cox regression model. For each index, the area under the receiver operating characteristic (ROC) curve (AUC) was calculated, and the clinical decision curve of each index was drawn. The clinical usefulness of the index was evaluated by decision curve analysis (DCA), which can quantify the net benefits at different threshold probabilities. The least absolute shrinkage and selection operator (LASSO) strategy (variables with a Wald Chi-square test result of *P* < 0.2 in the univariate Cox proportional hazards regression model were used as candidate factors), an efficient statistical method for coping with high-dimensional data^27^, was employed to screen the most useful predictive risk factors in Model 2 and the hypertension model. The optimal filtering of variables in the LASSO regression model was determined using tenfold cross-validation and the minimum criteria or one standard error of the minimum criteria (1SE criteria). Finally, Harrell’s C-index and the AUC of the prediction model combined with other risk factors were calculated in different sex and age groups (60 years was the boundary), and the maximum Youden index (sensitivity + specificity − 1) was used to explore the best prediction index of new-onset hypertension in different subgroups. Meanwhile, the area under the ROC curve (AUC) between 0.5 and 0.6 suggests poor accuracy of the diagnostic test. AUC between 0.6 and 0.7 suggests sufficient accuracy, between 0.7 and 0.8 good accuracy, and between 0.8 and 0.9 very good accuracy, whereas AUC higher than 0.9 suggests excellent accuracy of the diagnostic test^28^. The method used for missing data in other non-main research variables was multiple imputation. All tests were two-sided, and *P*-values < 0.05 were considered statistically significant. SPSS version 23.0 (IBM Corp., Armonk, NY, USA) and R software (version 3.6.3; R Foundation, Vienna, Austria) were used for the analyses.

### Patient and public involvement statement

The patients were not actively involved during the design and conduct of this study. The patients and the general public will be informed of the study results through peer-reviewed journals.

### Patient consent for publication

Consent obtained directly from patient(s).

### Ethical approval

This study was conducted according to the Helsinki Declaration of the World Medical Association and was approved by the Ethics Committee of China Medical University (Shenyang, China; AF-SDP-7-1, 0-01). All participants provided written informed consent.

## Results

### Baseline characteristics and Cox regression analysis

The baseline demographic characteristics and the results of the univariate Cox regression analysis of the 4123 eligible participants are shown in Table 1. The average age was 50.11 ± 9.40 years. Naturally, the cumulative incidence rate of new-onset hypertension increased with age (the rates for age < 40 and 40–60 were 12.39% and 19.33%, respectively). The highest incidence of new-onset hypertension was 28.92% (188/650) in people > 60 years old, and the risk of hypertension increased 1.84 times (95% CI 1.42–2.37) and 3.69 times (95% CI 2.78–4.91) when reaching 40 and 60 years old, respectively.

**Table 1:** Baseline characteristics and univariate cox regression analysis.

Variables	Total	New-onset hypertension		*P*-value	HR (95% CI)	*P*-value
	n = 4123	No (n = 3305)	Yes (n = 818)			
Age categorical (years, n, %)	50.11 ± 9.40	49.39 ± 9.12	53.03 ± 9.94	< 0.001		< 0.001
< 40	597 (14.48%)	523 (15.82%)	74 (9.04%)		1 (Reference)	
40–60	2876 (69.75%)	2320 (70.20%)	556 (67.97%)		1.84 (1.42, 2.37)	< 0.001
> 60	650 (15.76%)	462 (13.97%)	188 (22.98%)		3.69 (2.78, 4.91)	< 0.001
Male (n, %)	1746 (42.34%)	1323 (40.04%)	423 (51.71%)	< 0.001	1.25 (1.08, 1.45)	< 0.001
Current smoking (n, %)	1492 (35.51%)	1157 (35.01%)	335 (40.95%)	< 0.001	1.11 (0.96, 1.28)	0.174
Current drinking (n, %)	825 (20.01%)	610 (18.45%)	215 (26.28%)	< 0.001	1.19 (1.01, 1.41)	0.03
Marriage (n, %)				0.04		
Unmarried	37 (0.91%)	29 (0.88%)	8 (0.97%)		1 (Reference)	
Married	3904 (94.69%)	3144 (95.13%)	760 (92.90%)		1.06 (0.50, 2.22)	0.8879
Divorce	27 (0.65%)	17 (0.51%)	10 (1.22%)		1.54 (0.57, 4.12)	0.395
Widowed	155 (3.75%)	115 (3.47%)	40 (4.89%)		1.56 (0.70, 3.52)	0.279
Education (n, %)				0.03		
Illiteracy	272 (6.60%)	214(6.48%)	58 (7.09%)		1 (Reference)	
Primary school	1571 (38.10%)	1221 (36.94%)	350 (42.79%)		1.27 (0.95, 1.71)	0.109
Junior high school	1855 (44.99%)	1516 (45.87%)	339 (41.44%)		0.89 (0.66, 1.20)	0.444
High school and above	425 (10.31%)	349(10.56%)	76 (9.29%)		0.90 (0.63, 1.30)	0.583
Annual income (CNY, n, %)				0.757		
< 2000	113 (2.74%)	94 (2.84%)	19 (2.32%)		1 (Reference)	
2000–5000	2593 (62.91%)	2081(62.97%)	512 (62.34%)		1.39 (0.86, 2.26)	0.181
5000–20,000	1302 (31.58%)	1038 (31.41%)	264 (32.27%)		1.40 (0.86, 2.29)	0.178
> 20,000	115 (2.79%)	92 (2.78%)	23 (2.81%)		1.40 (0.75, 2.61)	0.297
Mean SBP (mmHg)	124.18 ± 9.53	123.00 ± 9.55	128.96 ± 7.79	< 0.001	1.06 (1.05, 1.07)	< 0.001
Mean DBP (mmHg)	75.01 ± 7.23	74.37 ± 7.10	77.62 ± 7.14	< 0.001	1.05 (1.04, 1.07)	< 0.001
Mean pulse (beats/min)	77.07 ± 12.05	77.13 ± 12.08	76.84 ± 11.92	0.561	0.99 (0.98, 1.00)	< 0.001
SBP mmHg (n, %)				< 0.001		
< 120	1238 (30.03%)	1132 (34.29%)	106 (12.96%)		1 (Reference)	
120–129	1477 (35.82%)	1205 (36.45%)	272 (32.25%)		2.01 (1.58, 2.54)	< 0.001
130–139	1408 (34.15%)	968 (29.29%)	440 (53.79%)		3.57 (2.86, 4.47)	< 0.001
DBP mmHg (n, %)				< 0.001		
< 70	890 (21.59%)	784 (23.72%)	106 (12.96%)		1 (Reference)	
70–79	2023 (49.07%)	1691 (51.16%)	332 (40.59%)		1.39 (1.11, 1.75)	0.004
80–89	1210 (29.35%)	830 (25.11%)	380 (46.45%)		2.56 (2.04, 3.21)	< 0.001
DM (n, %)	197 (4.77%)	135 (4.08%)	62 (7.57%)	< 0.001	1.76 (1.34, 2.32)	< 0.001
Fasting blood glucose (mmol/L)	5.61 ± 1.20	5.55 ± 1.12	5.82 ± 1.43	< 0.001	1.11 (1.06, 1.16)	< 0.001
High WBC (n, %)	96 (2.33%)	72 (2.18%)	24 (2.93%)	0.205	1.12 (0.73, 1.71)	0.607
eGFR (mL/min/1.73 m^2^, n, %)				< 0.001		
> 90	2852 (69.17%)	2336 (70.68%)	516 (62.08%)		1 (Reference)	
60–89	1246 (30.22%)	946 (28.62%)	300 (36.67%)		1.50 (1.29, 1.74)	< 0.001
< 60	25 (0.60%)	23 (0.69%)	2 (0.24%)		1.64 (0.68, 3.95)	0.274
High UA (n, %)	199 (4.83%)	140 (4.24%)	59 (7.21%)	0.001	1.73 (1.31, 2.29)	< 0.001
High TC (n, %)	507 (12.29%)	378 (11.44%)	129 (15.77%)	0.001	1.15 (0.95, 1.40)	0.158
High TG (n, %)	440 (10.67%)	336 (10.17%)	104 (12.71%)	0.051	1.29 (1.04, 1.60)	0.02
Low HDL-C (n, %)	493 (11.96%)	391 (11.86%)	102 (12.47%)	0.692	0.95 (0.76, 1.18)	0.657
High LDL-C (n, %)	214 (5.19%)	165 (4.99%)	49 (5.99%)	0.241	0.99 (0.73, 1.34)	0.949
Family history of diabetes (n, %)	521 (12.63%)	415 (12.56%)	106 (12.95%)	0.79	0.88 (0.71, 1.09)	0.231
Family history of hypertension (n, %)	807 (19.57%)	637 (19.27%)	170 (20.78%)	0.377	1.27 (1.06, 1.52)	0.008
Family history of CHD (n, %)	569 (13.80%)	449 (13.59%)	120 (14.67%)	0.448	1.23 (1.00, 1.51)	0.045
Family history of stroke (n, %)	605 (14.67%)	454 (13.73%)	151 (18.46%)	0.002	2.08 (1.72, 2.51)	< 0.001
Vegetables (per day, n, %)				0.264		
Rarely	74 (1.79%)	56 (1.69%)	18 (2.20%)		1 (Reference)	
250 g	302 (7.32%)	242 (7.32%)	60 (7.33%)		1.07 (0.61, 1.87)	0.807
250–500 g	1020 (24.74%)	826 (24.99%)	194 (23.72%)		0.98 (0.59, 1.64)	0.951
500–1000 g	1200 (29.11%)	981 (29.68%)	219 (26.78%)		0.95 (0.57, 1.59)	0.858
More than 1000 g	1527 (37.04%)	1200 (36.31%)	327 (39.98%)		1.40 (0.85, 2.31)	0.191
Meat (per week, n, %)				0.650		
Rarely	716 (17.37%)	563 (17.03%)	153 (18.70%)		1 (Reference)	
250 g	1058 (25.66%)	850 (25.72%)	208 (25.43%)		0.81 (0.65, 1.01)	0.056
250–500 g	1267 (30.73%)	1026 (31.04%)	241 (29.46%)		0.67 (0.54, 0.82)	< 0.001
More than 500 g	1082 (26.24%)	866 (26.20%)	216 (26.41%)		0.80 (0.65, 1.00)	0.046
Bean (per week, n, %)				0.311		
Rarely	1598 (38.75%)	1281 (38.76%)	317 (38.75%)		1 (Reference)	
2–3 times	2083 (50.52%)	1654 (50.04%)	429 (52.44%)		0.80 (0.69, 0.93)	0.0041
More than 4 times	442 (10.72%)	370 (11.19%)	72 (8.80%)		0.57 (0.44, 0.74)	< 0.001
Fat food (per week, n, %)				0.144		
Rarely	3281 (79.58%)	2628 (79.52%)	653 (79.83%)		1 (Reference)	
2–3 times	703 (17.06%)	571 (17.28%)	132 (16.14%)		0.86 (0.71, 1.05)	0.147
More than 4 times	138 (3.35%)	106 (3.21%)	32 (3.91%)		1.14 (0.80, 1.62)	0.464
Tea (per day, n, %)				0.06		
Never	2686 (65.14%)	2171 (65.69%)	515 (62.96%)		1 (Reference)	
Sometimes	834 (20.22%)	669 (20.24%)	165 (20.17%)		0.90 (0.75, 1.08)	0.259
Once above	603 (14.63%)	465 (14.07%)	138 (16.87%)		1.17 (0.96, 1.42)	0.118
Pickles (per week, n, %)				0.09		
Rarely	1476 (35.80%)	1207 (36.52%)	269 (32.88%)		1 (Reference)	
2–3 times	2142 (51.95%)	1686 (51.01%)	456 (55.75%)		1.16 (0.99, 1.36)	0.066
More than 4 times	505 (12.25%)	412 (12.46%)	93 (11.37%)		0.85 (0.66, 1.08)	0.186
Sleep duration (hours, n, %)				0.857		
< 6 h	592 (14.36%)	471 (14.25%)	121 (14.79%)		1 (Reference)	
6–8 h	2700 (65.48%)	2168 (65.36%)	532 (65.03%)		1.08 (0.88, 1.33)	0.459
> 8 h	831 (20.16%)	666 (20.15%)	165 (20.17%)		1.26 (0.99, 1.62)	0.062
Physical activity intensity (n, %)				0.076		
Light	1214 (29.44%)	989 (29.92%)	225 (27.51%)		1 (Reference)	
Moderate	776 (18.82%)	635 (19.21%)	141 (17.24%)		1.01 (0.81, 1.26)	0.941
Heavy	2132 (51.70%)	1677 (50.74%)	455 (55.62%)		1.47 (1.24, 1.74)	< 0.001
Physical exercise (n, %)	722 (17.51%)	568 (17.19%)	154 (18.83%)	0.338	1.05 (0.88, 1.27)	0.576
Snoring (n, %)	1353 (32.82%)	1029 (31.13%)	324 (39.61%)	< 0.001	1.37 (1.18, 1.59)	< 0.001

*SBP* systolic blood pressure, *DBP* diastolic blood pressure, *DM* diabetes mellitus, *WBC* leukocyte count, *eGFR* estimated glomerular filtration rate, *UA* uric acid, *TC* total cholesterol, *TG* total triglycerides, *HDL-C* high-density lipoprotein cholesterol, *LDL-C* low-density lipoprotein cholesterol, *CYN* Chinese yuan.

There was a significant difference in the baseline BP between the two groups with or without new-onset hypertension (*P* < 0.001). Male participants accounted for 42.34%, and men were more prone to developing high blood pressure than women (24.23% *vs*. 16.62%). Participants with lower education levels (junior high school and below) accounted for the majority (89.69%) of those with new-onset hypertension. There were differences in the education level and marital status between the two groups. Factors such as drinking status, smoking status, baseline BP, FBG, diabetes, snoring, family history of hypertension or stroke, low-frequency intake of bean products, hyperuricemia, hypercholesterolemia, and estimated glomerular filtration rate levels were significantly associated with the risk of new-onset hypertension, with statistical differences between the two groups.

### Obesity-related anthropometric characteristics

BRI, ABSI, BMI, WHR, WC and WHtR were statistically different between participants with and without new-onset hypertension in the whole population (data not shown). The participants were divided into four subgroups according to the two variables of age (60 years old) and sex. Table 2 shows the characteristics of obesity-related anthropometric indicators of participants according to sex and age. There was no significant difference between the obesity indexes of the two groups (*P* > 0.05) among women aged > 60 when the indices were analyzed as continuous variables. Among older men, the difference was not significant for BMI and ABSI between the group who progressed to hypertension and the non-hypertension group (*P* > 005). Individuals with hypertension had higher BMI, BRI, WHtR, ABSI, and WHR than those with no high blood pressure, regardless of sex.

**Table 2:** Characteristics of obesity-related anthropometric indicators of participants according to sex and age.

Male	Age: ≤ 60			Age: > 60		
New-onset hypertension	No (n = 1109)	Yes (n = 321)	*P*-value	No (n = 214)	Yes (n = 102)	*P*-value
WC (cm)	81.163 ± 9.113	83.516 ± 9.126	< 0.001	78.779 ± 8.622	81.308 ± 10.282	0.032
WHtR	0.486 ± 0.055	0.498 ± 0.055	< 0.001	0.479 ± 0.056	0.494 ± 0.057	0.045
WHR	0.852 ± 0.076	0.866 ± 0.068	0.003	0.844 ± 0.080	0.872 ± 0.074	0.005
BMI (kg/m^2^)	24.151 ± 3.543	24.836 ± 3.240	0.003	22.900 ± 2.986	23.335 ± 3.444	0.279
ABSI	0.075 ± 0.004	0.076 ± 0.006	0.041	0.076 ± 0.006	0.078 ± 0.005	0.063
BRI	3.154 ± 1.061	3.378 ± 1.016	0.001	3.027 ± 1.017	3.292 ± 1.091	0.047

Female	Age: ≤ 60			Age: > 60		
New-onset hypertension	No (n = 1734)	Yes (n = 309)	*P*-value	No (n = 248)	Yes (n = 86)	*P*-value
WC (cm)	77.751 ± 8.943	81.279 ± 8.955	< 0.001	78.324 ± 9.625	79.971 ± 9.925	0.198
WHtR	0.496 ± 0.057	0.521 ± 0.057	< 0.001	0.512 ± 0.065	0.522 ± 0.065	0.24
WHR	0.824 ± 0.074	0.849 ± 0.065	< 0.001	0.844 ± 0.072	0.854 ± 0.071	0.281
BMI (kg/m^2^)	23.969 ± 3.471	25.045 ± 3.549	< 0.001	23.230 ± 4.180	23.743 ± 4.125	0.349
ABSI	0.075 ± 0.005	0.076 ± 0.005	< 0.001	0.078 ± 0.007	0.079 ± 0.005	0.802
BRI	3.335 ± 1.086	3.806 ± 1.131	< 0.001	3.651 ± 1.275	3.853 ± 1.252	0.226

*WHR* waist hip ratio, *WC* waist circumference, *BRI* body roundness index, *WHtR* waist height ratio, *ABSI*, a body shape index, *BMI* body mass index.

### Clinical usefulness of anthropometric indices related to obesity

**Figure 2:**
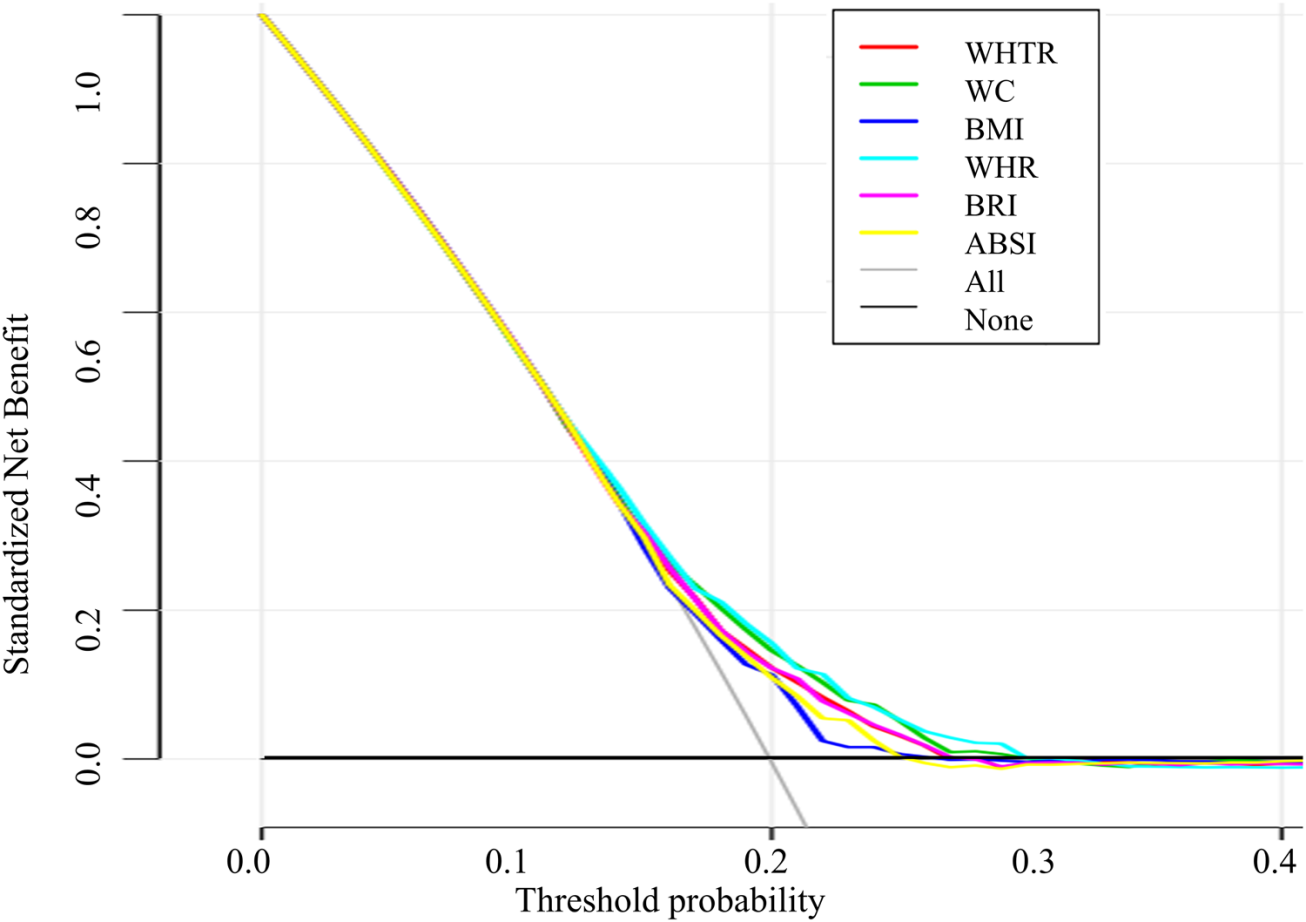
Area under the receiver operating characteristic (ROC) curve (AUC) of each obesity index in new-onset hypertension. *WHR* waist hip ratio, *WC* waist circumference, *BRI* body roundness index, *WHtR* waist height ratio, *ABSI* a body shape index, *BMI* body mass index.

**Figure 3:**
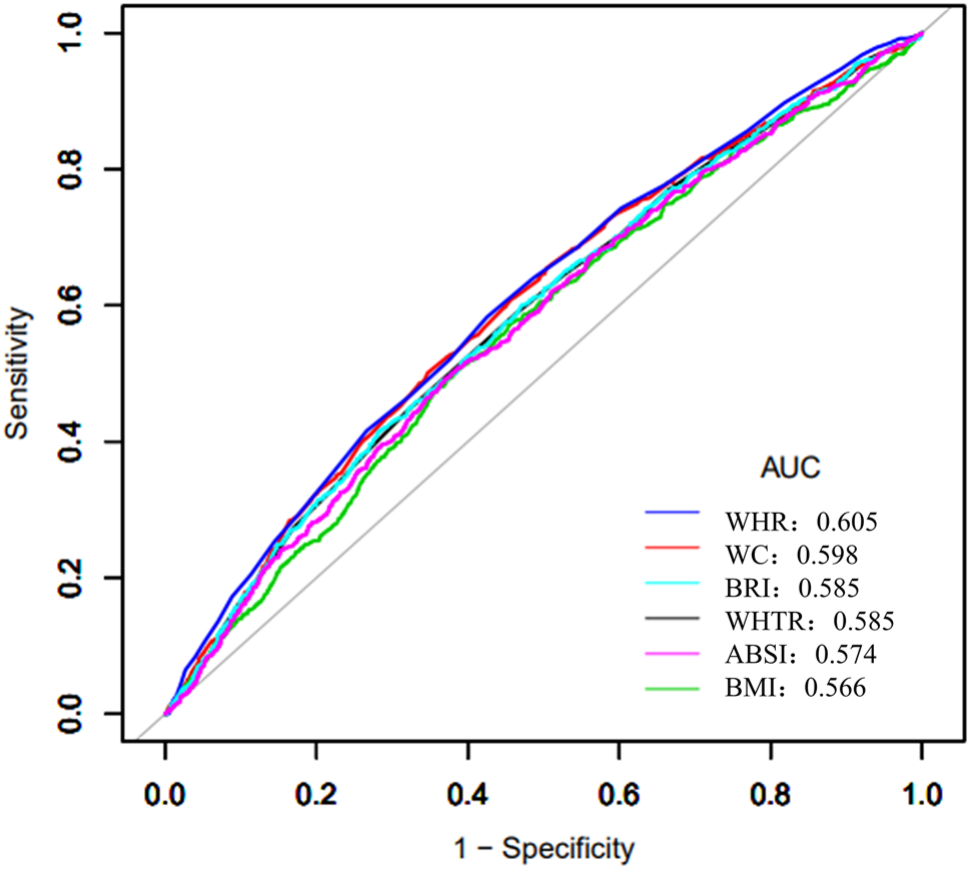
Decision curve analysis for the obesity indices. The x- and y-axes measure the threshold probability and standardized net benefit, respectively. The horizontal black line represents the hypothesis that none of the general population will receive an intervention. The grey solid line represents the hypothesis that interventions are offered to all participants. The other 6 lines represent the net benefits of offering interventions according to the threshold probabilities derived from the 6 obesity indices. *WHtR* waist height ratio, *WC* waist circumference, *BMI* body mass index, *WHR* waist hip ratio, *BRI* body roundness index, *ABSI* a body shape index.

The ROC curve (Fig. 2) shows the preliminary exploration of the predicted values of obesity-related anthropometric indices for new-onset hypertension. The curve suggests that WHR and WC have a better predictive value, with AUCs of 0.605 and 0.598, while the AUCs of ABSI and BRI were 0.574 and 0.585, respectively, which were not better than the conventional WHR. Moreover, the predictive ability of BMI was the poorest, with an AUC of 0.566. Figure 3 shows the DCA curves of new-onset hypertension within 2.5 years, suggesting that both conventional and unconventional obesity indices can improve the identification of new-onset hypertension. In terms of hypertension occurrence in the general population within the prediction threshold range of 15–25%, the prediction of BP intervention management according to both conventional and unconventional obesity indices was better than the “treat-all-patients” or “no-treatment” approaches. Overall, the standardized net benefit of the WHR was the greatest. Considering that the incidence of hypertension in the cohort over the 2.5 years was 19.84%, we suggest a threshold probability of 20% for the participants. The DCA curve also shows that WHR and WC may have the best potential predictive benefits.

### Subgroup analysis

The relationships between obesity-related indices and the risk of new-onset hypertension are shown in Table 3 (subgroup analysis based on sex and age). In the Cox regression analysis, Model 1 was a crude model without any adjustment, while Model 2 was adjusted for risk factors, including SBP, DBP, pulse, diabetes, family history of hypertension, family history of stroke, intake frequency of the beans, physical activity intensity, hyperuricemia, hypertriglyceridemia, and snoring. Among the men ≤ 60 years, WHtR, WHR, ABSI, and BRI were associated with an increased risk of hypertension (even after adjustment for other risk factors in Model 2). WHR was associated with the highest risk of hypertension with an HR of 2.28 (95% CI 1.73–3.01), while WC in men > 60 years was associated with the highest risk of hypertension, and the HR was 3.24 (95% CI 1.48–7.08). Regardless of age, WC, WHtR, WHR, ABSI, and BRI were positively correlated with the risk of hypertension in women. The HRs of WHR were the highest in all age groups (HR 2.38 in the ≤ 60 years age group; HR: 2.51 in the > 60 years age group). However, BMI did not predict the risk of new-onset hypertension regardless of sex and age in Model 2.

**Table 3:** Subgroup analysis of the risk of new-onset hypertension.

Male	Model 1	Model 2	Model 1	Model 2
	Age: ≤ 60		Age: > 60	
	HR (95% CI) *P*		HR (95% CI) *P*	
WC (> 90 cm)	1.89 (1.46, 2.44) < 0.001	1.25 (0.92, 1.71) 0.155	1.73 (1.04, 2.88) 0.035	3.24 (1.48, 7.08) 0.003
WHtR (> 0.5)	1.67 (1.33, 2.10) < 0.001	1.43 (1.09, 1.86) 0.008	1.62 (1.07, 2.45) 0.023	1.85 (1.03, 3.32) 0.038
WHR (> 0.9)	2.52 (1.98, 3.21) < 0.001	2.28 (1.73, 3.01) < 0.001	2.85 (1.83, 4.45) < 0.001	2.45 (1.34, 4.49) 0.003
BMI > 28 (kg/m^2^)	1.46 (1.07, 1.99) 0.018	0.99 (0.68, 1.44) 0.951	1.34 (0.62, 2.92) 0.459	2.88 (0.91, 9.10) 0.071
ABSI (High vs. low percentile)	1.82 (1.45, 2.30) < 0.001	1.61 (1.25, 2.08) < 0.001	2.21 (1.38, 3.52) < 0.001	1.82 (1.02, 3.25) 0.041
BRI (High vs. low percentile)	1.67 (1.33, 2.10) < 0.001	1.43 (1.10, 1.86) 0.007	1.60 (1.06, 2.42) 0.026	1.85 (1.03, 3.30) 0.037

Female	Age: ≤ 60		Age: > 60	
	HR (95% CI) *P*		HR (95% CI) *P*	
WC (> 80 cm)	2.23 (1.76, 2.83) < 0.001	2.02 (1.57, 2.62) < 0.001	1.39 (0.89, 2.18) 0.144	1.81 (1.01, 3.24) 0.047
WHtR (> 0.5)	2.37 (1.84, 3.05) < 0.001	2.06 (1.57, 2.70) < 0.001	1.35 (0.84, 2.15) 0.216	1.86 (1.03, 3.36) 0.038
WHR (> 0.85)	2.66 (2.10, 3.37) < 0.001	2.38 (1.84, 3.09) < 0.001	1.94 (1.24, 3.05) 0.004	2.51 (1.43, 4.41) 0.001
BMI > 28 (kg/m^2^)	1.54 (1.13, 2.09) 0.005	1.16 (0.82, 1.64) 0.390	1.17 (0.58, 2.36) 0.654	0.56 (0.21, 1.45) 0.232
ABSI (high vs. low percentile)	2.23 (1.76, 2.83) < 0.001	2.19 (1.70, 2.83) < 0.001	1.67 (1.01, 2.79) 0.047	2.16 (1.17, 3.98) 0.013
BRI (high vs. low percentile)	2.49 (1.93, 3.22) < 0.001	2.19 (1.66, 2.88) < 0.001	1.37 (0.85, 2.20) 0.191	1.82 (1.01, 3.31) 0.048

Model 1: Crude Model; Model 2: adjusted for SBP, DBP, pulse, diabetes, family history of hypertension, family history of stroke, intake frequency of the beans, physical activity intensity, hyperuricemia, hypertriglyceridemia, and snoring.

*HR* Hazard ratio, *CI* confidence interval, *WHR* waist hip ratio, *WC* waist circumference, *BRI* body roundness index, *WHtR* waist height ratio, *ABSI* a body shape index, *BMI* body mass index, *SBP* systolic blood pressure, *DBP* diastolic blood pressure.

### Comparison of predicted values

Table 4 shows the comparison of predicted values of obesity indicators based on different sex and age groups. We compared the AUCs and C-indices of the hypertension prediction models constructed by obesity-related indices with age, SBP, DBP, pulse, diabetes, family history of hypertension, family history of stroke, intake frequency of the beans, physical activity intensity, hyperuricemia, hypertriglyceridemia, and snoring. To avoid over-filling the constructed prediction models and possible collinearity between variables, the screening of risk factors included the variables screened by LASSO analysis and conventional independent predictors of hypertension. Among the obesity-related anthropometric indices of women in the two age groups, WHR was accompanied by the largest AUCs (0.793 in the ≤ 60 years age group and 0.716 in the > 60 years age group) and C-indices (0.776 and 0.696, respectively). In addition, WC (AUC = 0.759, C-index = 0.746) and WHR (AUC = 0.788, C-index = 0.721) were the best predictors of new-onset hypertension in men ≤ 60 and > 60 years, respectively.

**Table 4:** Comparison of the predicted value of obesity indicators based on different sex and age groups.

Variable	AUC	C-index (95% CI)	Sensitivity	Specificity	Youden index
Male age: ≤ 60					
WC (cm) + other factors	0.759	0.746 (0.708–0.782)	0.781	0.614	0.395
WHtR + other factors	0.748	0.736 (0.704–0.769)	0.815	0.553	0.368
WHR + other factors	0.753	0.743 (0.711–0.775)	0.760	0.617	0.377
BMI (kg/m^2^) + other factors	0.743	0.731 (0.698–0.764)	0.683	0.683	0.366
ABSI + other factors	0.749	0.742 (0.709–0.775)	0.669	0.690	0.359
BRI + other factors	0.748	0.736 (0.703–0.769)	0.820	0.547	0.367
Male age: > 60					
WC(cm) + other factors	0.744	0.721 (0.657–0.784)	0.681	0.713	0.394
WHtR + other factors	0.728	0.721 (0.659–0.781)	0.667	0.701	0.368
WHR + other factors	0.788	0.743 (0.688–0.797)	0.617	0.835	0.452
BMI (kg/m^2^) + other factors	0.729	0.727 (0.669–0.784)	0.788	0.579	0.367
ABSI + other factors	0.784	0.741 (0.685–0.798)	0.688	0.689	0.377
BRI + other factors	0.730	0.721 (0.660–0.781)	0.778	0.595	0.373
Female age:** ≤ **60					
WC (cm) + other factors	0.777	0.767( 0.734–0.798)	0.674	0.749	0.423
WHtR + other factors	0.774	0.768 (0.736–0.800)	0.702	0.721	0.423
WHR + other factors	0.793	0.776 (0.745–0.808)	0.677	0.749	0.426
BMI (kg/m^2^ ) + other factors	0.769	0.755 (0.722–0.789)	0.694	0.712	0.406
ABSI + other factors	0.779	0.776 (0.745–0.806)	0.674	0.749	0.423
BRI + other factors	0.769	0.767 (0.735–0.799)	0.674	0.749	0.423
Female age: > 60					
WC (cm) + other factors	0.685	0.677 (0.600–0.716)	0.543	0.760	0.303
WHtR + other factors	0.679	0.664 (0.588–0.739)	0.524	0.781	0.305
WHR + other factors	0.716	0.696 (0.629–0.763)	0.661	0.659	0.32
BMI (kg/m^2^) + other factors	0.683	0.665 (0.589–0.741)	0.524	0.773	0.297
ABSI + other factors	0.691	0.669 (0.593–0.745)	0.592	0.727	0.319
BRI + other factors	0.683	0.665 (0.589–0.740)	0.533	0.772	0.305

Other factors: SBP, DBP, pulse, diabetes, family history of hypertension, family history of stroke, intake frequency of the beans, physical activity intensity, hyperuricemia, hypertriglyceridemia, snoring, and menopause (for women).

*CI* confidence interval, *WHR* waist hip ratio, *WC* waist circumference, *BRI* body roundness index, *WHtR* waist height ratio, *ABSI* a body shape index, *BMI* body mass index, *SBP* systolic blood pressure, *DBP* diastolic blood pressure, *AUC* area under ROC curve, *ROC* receiver operating characteristic.

## Discussion

In this study, we supplemented the predictive values of the unconventional obesity-related indices ABSI and BRI when forecasting hypertension. At the same time, we compared the predictive values of obesity-related anthropometric indices among Chinese adults divided into four groups according to sex and age for predicting the incidence of hypertension, as well as exploring the best predictive indicators. By using the survey data of a prospective cohort of general people in rural areas of northeast China, we found strong evidence of a positive correlation between obesity-related anthropometric indicators and the risk of hypertension. During the median follow-up of 2.59 years, 818 (19.8%) new cases of hypertension occurred. Unconventional obesity indices BRI and ABSI had predictive value for new-onset hypertension, but they were not better than conventional indices, and BMI had limited predictive value. The best predictor of hypertension in women from the two age groups was WHR, while WC and WHR were the best predictors of new-onset hypertension in men ≤ 60 and > 60 years, respectively. Our results highlight the obesity index is most closely related to new-onset hypertension in the short term in different sex and age groups. This will benefit primary health care regarding the control of and monitoring of the incidence of hypertension.

We also found that compared with other obesity indices, WHR and WC have a greater impact on the risk of hypertension in the general population. ABSI and BRI also have some predictive values as unconventional obesity anthropometric indicators. These findings are consistent with previous ultrasound studies, which found that visceral fat increases the risk of hypertension^29^. Excessive visceral fat distribution is often accompanied by changes in inflammation levels, hormones, and endothelial cells (including decreased adipocytokines, increased free fatty acids, uric acid, leptin, and vascular endothelial growth factor)^30^. Obesity can raise BP, and is reported to cause 65–75% of essential hypertension cases^31^. Obesity-related hypertension may be the result of one or more causes. It may lead to increased BP levels through activation of the renin–angiotensin–aldosterone system (RAAS), overactivation of the sympathetic nervous system, renal sodium reabsorption, and endothelial dysfunction, and increased insulin resistance^32^. These obesity indicators may be associated with visceral obesity. Previous studies in Asia found that WC and WHR were the most useful indicators for identifying adult diabetes and hypertension in South Asia^33,34^.

For WC, the rising trend of global obesity is significant. From 2013 to 2018, the average WC of men increased from 82 to 86.3 cm, and that of elderly women increased from 79.1 to 83.4 cm^35^. Most studies^36^ found a positive correlation between WC and BP, but not in a prospective study of a European population^29^. The differences in results may be due to ethnic differences, low participation rates, and relatively high withdrawal rates in the study. Previous studies in Iran found WC had a higher predictive value for hypertension than BMI in women aged 40–60, which was similar to our conclusion^37^. WHR and WC do not consider height. However, they can better reflect increases in visceral fat. Therefore, they may be preferred obesity indices of predicting hypertension in rural China. For BMI, given the significant differences in different regions, races, and body shapes, the use of a unified range may underestimate or overestimate the harm of the disease. Chinese people are relatively thin, and a previous study found that a BMI > 25 kg/m^2^ was associated with elevated BP^10^. Therefore, this study’s BMI standard (28 kg/m^2^) was relatively lower than European and American standards to avoid underestimation of obesity. Nevertheless, the predictive value of BMI for new-onset hypertension is relatively limited. This may be attributed to its inability to distinguish between muscle and fat; thus, it cannot reflect the actual degree of obesity, which is consistent with previous studies^12^.

In terms of subtle sex differences, menopause is a well-known risk factor for hypertension. The sudden decline in the circulating estrogen level^38^ may independently lead to high BP through some unknown mechanisms, such as the direct effect on the arterial wall, RAAS activation, and the sympathetic nervous system^39^. Considering that the baseline average age of women in the analysis cohort was 49.55 years, most of them had reached a menopausal state. The menopausal state was added as an adjustment factor in the female population. However, menopausal status and premature menopause (menopause < 45 years old) were not found to increase the risk of hypertension in this study cohort, which may be due to the relatively short follow-up time or relatively young age.

Our study has the following advantages. First, a prospective cohort study can fully demonstrate causality. Second, to the best of our knowledge, this is the first time a study has compared the predictive value of conventional and unconventional new obesity indicators for new-onset hypertension and explored the best predictive indicators in different sex and age groups; this will be conducive to the primary prevention of hypertension in primary medical and health institutions. Third, this study included a relatively comprehensive health screening program based on the general rural population, including routine blood biochemistry, physical measurement, diseases and demographic information, physical activities, and daily living habits. However, this study also has some limitations. First, the follow-up time was short. Second, our conclusions may only be applicable to the economically underdeveloped northeast rural areas in Asia. Due to the relatively thin body shape characteristics of Asians, the selection of predictive indicators may not be applicable to other races, and the extrapolation is limited. Third, given the low incidence of secondary hypertension in the general population, although the type of new-onset hypertension was further determined and verified in the follow-up (no suspicious secondary hypertension events were identified through the available information, such as renal function ion and symptom description), we did not use a gold standard, such as renal ultrasound, angiography, and respiratory and sleep detection.

## Conclusions

In middle-aged and elderly populations, ABSI and BRI are independently and positively correlated with the risk of new-onset hypertension. Notably, different best predictors of hypertension exist in different age and sex subgroups. Women and men over the age of 60 should pay attention to WHR, and men under the age of 60 should pay attention to WC. Therefore, an in-depth subgroup analysis will help to design effective primary prevention strategies for specific middle-aged and elderly groups.

## Data Availability

Only the data underlying this article are available for validation analysis and will be shared by the corresponding author on reasonable request; no additional, related documents will be shared.
